# Clinical and Laboratory Outcomes in HIV-1 and HTLV-1/2 Coinfection: A Systematic Review

**DOI:** 10.3389/fpubh.2022.820727

**Published:** 2022-03-07

**Authors:** Iris Montaño-Castellón, Cleyde Sheyla Chachaqui Marconi, Clara Saffe, Carlos Brites

**Affiliations:** ^1^Laboratório de Pesquisa em Infectologia (LAPI), Hospital Universitário Professor Edgard Santos (HUPES), Salvador, Brazil; ^2^Programa de Pós Graduação em Medicina e Saúde (PPgMS), Universidade Federal da Bahia (UFBA), Salvador, Brazil; ^3^Escola Bahiana de Medicina e Saúde Pública (EBMSP), Salvador, Brazil

**Keywords:** HIV-1, HTLV-1, HTLV-2, coinfection, clinical outcomes, mortality, survival

## Abstract

**Aim:**

To perform a systematic review to describe the available findings on clinical outcomes in HIV-1 and HTLV-1/HTLV-2 co-infected individuals since 1995.

**Design:**

This Systematic Review used PECO criteria follow by PRISMA reporting guidelines and registered as CRD42021279062 (Prospero database). The Newcastle-Ottawa Scale assessed the methodological quality of included studies.

**Data Collection and Analysis:**

A systematical search in PubMed/MEDLINE, Embase, Web of Sciences databases for cross-sectional, case-control, or cohort studies design to identify clinical and laboratorial outcomes related to HIV-1 and HTLV-1/2 coinfection. Search strategy: [(“HIV-1” AND “HTLV-1” OR “HTLV-2”) AND (“Coinfection”) AND (1990/01/01:2021/12/31[Date- Publication])].

**Results:**

A total of 15 articles were included on this systematic review describing data of 2,566 mono and coinfected patients, 58% male, with mean age was 35.7 ± 5.7 years. HIV-1 and HTLV-1 coinfected patients were more likely to had shorter survival and faster progression to death or mortality than monoinfected ones. Coinfected had higher CD4 cell counts and less likelihood of ART use. In addition, higher frequency of diseases like ichthyosis (22.2 vs. 6.8%), scabies (18.6 vs. 0%), candidiasis (42 vs. 12%), Strongyloidiasis (15.4 vs. 2%) and neurological manifestations like encephalopathy, peripheral neuropathy and HAM/TSP were more frequently reported in coinfected patients.

**Conclusions:**

HIV-1 and HTLV-1 coinfection and HIV-1 and HTLV-1 /2 triple coinfection were related to shorter survival, higher mortality rate, and faster progression to death, while coinfection by HIV-1/HTLV-2 seems to have neutral association with longer survival, slower AIDS progression, and lower mortality rate. The available evidence indicates an urgent need for prevention and control measures, including screening, diagnosis, and treatment of HIV-1 and HTLV-1/2 coinfected patients. Test-and-treat strategy for patients living with HIV in areas endemic for HTLV infection is mandatory, to avoid the risks of delayed therapy and death for coinfected patients.

**Systematic Review Registration:**

https://www.crd.york.ac.uk/prospero/, identifier: CRD42021279062.

## Introduction

Human T-cell lymphotropic virus (HTLV) was the first human retrovirus described (1). There are four types of HTLV but only two (HTLV-1 and HTLV-2) are associated with diseases. HTLV-1 is the causative agent of adult T-cell leukemia and tropical spastic paraparesis, while HTLV-2 has been associated with peripheral neuropathy and potentially with tropical spastic paraparesis (2). The human immunodeficiency virus (HIV) causes a progressive depletion of T cells that leads to severe immunodeficiency, increasing the risk of opportunistic infections and malignant neoplasms (3). HIV and HTLV belong to the same family (Retroviridae), share genomic organization, tropism for CD4+ and CD8+ T cells, and routes of infection (sexual, parenteral and vertical). In consequence of common routes of infection, coinfection by both viruses is frequently detected in endemic areas, with higher prevalence in large metropolitan areas (4, 5).

HIV-1 and HTLV-1 infect the same cells but have different biological characteristics (6). This can explain why coinfection by these viruses is able to modify the natural history of both infections. In HIV-1coinfection by HTLV-1 differs from that by HTLV-2 in regulating cellular activation of target cells: HTLV-1 promotes a high level of cellular activation, while it is lower in HTLV-2 coinfected subjects. Both viruses promote increase in the frequency of CD4+ cells, but it does not result in an evident benefit to the immune response (4, 7).

Some studies have identified that HIV-1 subjects coinfected by HTLV are at higher risk of developing neurological complications, especially HAM/TSP (Human T-lymphotropic virus type-I-associated myelopathy/tropical spastic paraparesis), Adult T-cell leukemia (ATL), neuropathies, opportunistic infections, accelerated progression of HIV, and shorter survival (4, 8). On the other hand, some studies support the notion that co-infection by HIV-1 / HTLV-2 does not alter the clinical course of disease, or even that they can promote a protective effect (9–11). Available studies on coinfection are heterogeneous and fail to identify the factors driving the outcomes observed in this population. The aim of this review is to describe the available findings on clinical and laboratory outcomes in HIV-1 and HTLV-1 or HTLV-2 co-infected individuals since 1995.

## Materials and Methods

Preferred Reporting Items for Systematic Reviews and Meta-Analyses (PRISMA) reporting guidelines were followed (12), and our systematic review protocol was registered in the International Prospective Register of Systematic Reviews (PROSPERO, register number: CRD42021279062).

### Eligibility Criteria

Studies considered for inclusion were those with a cross-sectional, case-control, or cohort design reporting clinical outcomes: AIDS progression, death, mortality, survival, comorbidities, and opportunistic diseases in a population of HIV-1 and HTLV-1 or HTLV-2 coinfected individuals of all ages, regardless gender, race or ethnicity.

### Search Strategy

A systematical search in Pubmed/MEDLINE, Embase, Web of Sciences databases was done in August 2021, for articles published from 1990 to 2021, without language restriction. Our search strategy included three principal clusters of terms, one related to HIV-1 infection, another one to HTLV-1 or HTLV-2 infection and the last one about coinfection. The following terms to search PubMed/MEDLINE were:

((“HIV”[Mesh] OR “HIV Infections”[Mesh] OR “HIV-1”[Mesh] OR “HIV-1”[Title/Abstract] OR “HIV-I”[Title/Abstract]) **AND** (“Human T-lymphotropic virus 1”[Mesh] OR “HTLV-I Infections”[Mesh] OR “HTLV-II Infections”[Mesh] OR “HTLV-1”[Title/Abstract] OR “HTLV-2”[Title/Abstract] OR “HTLV-I”[Title/Abstract] OR “HTLV-II”[Title/Abstract]) **AND** (“Coinfection*”[Mesh] OR “Coinfection*”[Title/Abstract] OR “Co-infection*”[Title/Abstract] OR “coinfected”[Title/Abstract]) **AND** (1990/01/01:2021/12/31[Date - Publication])).

### Study Selection and Data Extraction

This review included research articles reporting clinical outcomes in HIV-1 and HTLV-1 or HTLV-2 coinfected individuals. Three reviewers screened the eligible articles in a blinded form using Rayyan tool to read the titles and abstracts, and using the PECO (Population, Exposition, Control and Outcomes) criteria (Table 1), to identify only the studies that met the objectives of the review (13). The studies identified as relevant by title and abstract reading (*n* = 33) were read in full and 15 studies were eligible for this review. Any divergence between the researchers was resolved by sending the conflicting article to a fourth reviewer, and disagreements were solved by discussion among the reviewers. The elimination of duplicates and the full text read stage were done using the Mendeley reference manager. Evaluation of the methodological quality of the studies was assessed by The Newcastle-Ottawa Scale (NOS) and one version adapted for cross-sectional studies (14).

**Table 1:** The PECO criteria used in the study.

**Criteria**	**Description**
Participants	Inclusion criteria: HIV 1 and HTLV1/2 coinfected individuals of all ages, without regard to gender, race, or ethnicity. Exclusion criteria: HTLV 1 or HTLV 2 monoinfection, HIV-2 coinfection, triple non-HIV/HTLV coinfections.
Exposure	Coinfection (HIV-1 and HTLV 1/2).
Control	Due to limited number of studies and their methodological type (a control group was not mandatory). However, data from HIV-1 mono-infected patients, when available, will be included for comparison's purposes.
Outcomes	Clinical outcomes related to HIV 1 and HTLV 1/2 coinfection defined as one of the following outcomes: opportunistic diseases, AIDS progression, death, mortality, survival, and comorbidities. Laboratorial: CD4 cell count. ART use.

*HIV, human immunodeficiency virus; HTLV, human T-cell lymphotropic virus; AIDS, acquired immunodeficiency syndrome*.

## Results

### Included Studies

**Figure 1:**
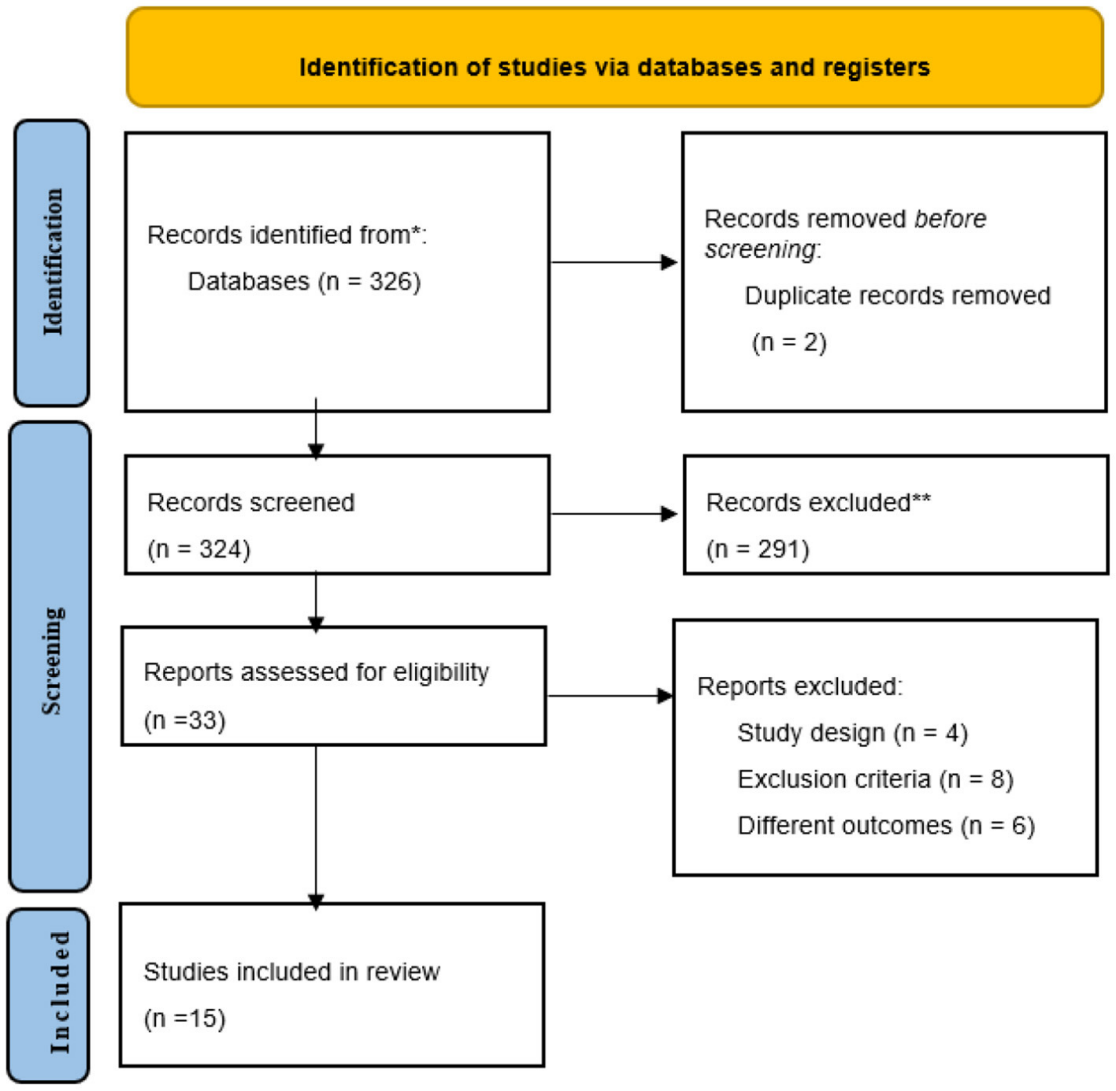
PRISMA 2020 Structured search strategy flow diagram.

A total of 326 articles were identified by the search strategy as possibly relevant for the systematic review. After duplicates were removed (*n* = 2), 324 articles were eligible for title and abstract reading. By using PECO criteria as described in Table 1, were selected 33 articles for full-text reading, 18 of them were excluded. Finally, 15 articles were considered to this systematic review and included 5 case control 6 cross-sectional, and 4 cohort studies as shown in Figure 1.

### Quality Assessment

The risk of bias in the study was rated as “low” by using NOS scale and an adaptation for cross sectional studies The mean final scores were 7 (case-control studies), 6 (cross sectional studies), and 6.5 (cohort studies).

### Patients Characteristics

The data of 2,566 mono and coinfected patients were described, 58% male. Mean age was 35.7 ± 5.7 years, only one study included children (2–16 years). Two studies did not report mean age of patients but informed that most participants were aged >35 or <40 years. A total of 581 individuals were coinfected, as follows: 319 (HIV-1/HTLV-1), 178 (HIV-1/ HTLV-2), and 84 (HIV-1/ HTLV-1/2), as shown in Table 2.

**Table 2:** Summary of the included studies related to HIV-1 and HTLV-1/2 coinfection.

**Study**	**Study Design, country**	**Population Characteristics**	**Coinfection**	**Main outcomes: Coinfected vs. Monoinfected**	**Other outcomes**
Page (15)	Cohort, USA	Total *n* = 107 Male: 78 (72.9%) Age >35: *n* = 58	HTLV-1/2 and HIV-1, *n* = 23	Coinfected patients had shorter survival, [HR: 3.326; CI 95% (1.12–9.87)] and were more likely to die of AIDS [RR: 2.92, CI 95% (1.30–6.95)].	Death was mainly due to respiratory impairment involving either *Pneumocystis carinii* pneumonia or other opportunistic infections in the lungs.
Kaplan (16)	Cross- sectional, USA	Total *n* = 184 Male: 127 (85.1%) Age <40 *n* = 144	HTLV-2 and HIV-1, *n* = 36	Ichthyosis: 8/36 (22.2%) vs. 12/148 (6.8%), *p* = 0.038.	Clinical presentation of coinfected patients was far from usual acquired ichthyosis: the scales were large, rhomboidal, and extremely coarse, resembling those of lamellar ichthyosis and frequent palmoplantar keratoderma.
Visconti (11)	Cohort, Italy	Total *n* = 145 Male: 103 (71%) Age: m = 28.8 (19–41)	HTLV-2 and HIV-1, *n* = 22	Survival was longer for coinfected patients (*p* = 0.08). No difference related to AIDS progression (*p* = 0.10) or death (*p* = 0.30) was found.	When the analysis was restricted to (IV A) or (IV) stage, no survival-related difference was found, *p* = 0.5.
Schechter (7)	Retrospective nested, case control, Brazil	Total *n* = 126 Male: 90 (71.4%) Age: m = 38.2 (±9.2)	HTLV-1 and HIV-1, *n* = 27	AIDS progression:4(15%) vs. 3(3%), [OR: 4.9; 95% CI (1.1–21.9)]. WHO late clinical stages (3 and 4): 12(44%) vs. 21 (21%), [OR: 3.1; 95% CI (1.2–7.8)].	CD4+ T cell count: 21 (88%) of coinfected patients had a greater count than 0.200 X109 cells/ L vs. 52 (56%) of monoinfected ones [OR: 4.0; 95% CI (1.3–12.5)]. Only 5% of monoinfected and none of coinfected (0%) were on ART, *p* = 0.58.
Giacomo (17)	Cross- sectional, Italy	Total *n* = 49 Male: 33 (67%) Age: m = 32.4 (±4.1)	HTLV-2 and HIV-1, *n* = 5	Progression to AIDS and death from AIDS was slower in coinfected subjects, *p* = <0.01, no difference was found in the cumulative survival estimated by Cox test. *p* = 0.23.	Higher CD4cell count at 36–47 months for coinfected 625 ± 231 vs. 390 ± 231 for monoinfected ones, *p* = 0.23.
Hershow (18)	Cohort, USA	Total *n* = 370 Female:101(27.3%) Age: m = 31.4 (± 8.6)	HTLV-2 and HIV-1, *n* = 61	Coinfection was not associated with AIDS or AIDS progression, Univariate RH: 0.82, 95% CI (0.34–1.94); Multivariate RH: 0.74, 95% CI (0.28–1.97).	Coinfected patients had a higher CD4 cells median at the time of death: 113 vs. 10 cells, *p*= 0,023.
Brites (8)	Case-Control, Brazil	Total *n* = 198 Male: 75% Age: 32.6 (m = 33)	HTLV-1 and HIV-1, *n* = 63	Coinfected patients had a shorter survival than monoinfected ones: 1,849 vs. 2,430 days, *p* = 0.001.	Patients who died of AIDS had an initial CD4 cell count similar to survivors: 371 ± 244 vs. 481 ± 417 cells/mm^3^, *p* = 0.7
Brites (19)	Cross-sectional, Brazil	Total *n* = 91 Female: 52(57.1%) Age: m = 36.25 (±16.45)	HTLV-1 and HIV-1, *n* = 18	Higher mortality rate among coinfected patients: *n* = 5, *p* = 0.01.	Severe forms of scabies are strongly associated with HTLV-1 infection OR: 3.0; 95% CI (1.85–4.86). Crusted form was highly predictive of coinfection (*p* = 0.01). All deaths were on coinfected, presented with crusted scabies and with a deeper degree of immunodeficiency.
Zehender (20)	Cohort, Italy	Total *n* = 90 Male: 64 (71%) Age:m = 32.5 (23-55)	HTLV-2 and HIV-1, *n* = 30	Higher probability of developing PN in coinfected than in monoinfected patients, *p* = 0.004.	None of the patients with PN were on ART when the symptoms appeared. ART use in coinfected individuals was less frequent 16 (53.3%) compared with monoinfected 49 (81.6%). AIDS progression rate was not different between groups, *p* = 0.1
Castro-Sansores (21)	Prospective cross- sectional, Mexico	Total *n* = 192 Male: 149(78%) Age: m = 32.7 (17–75)	HTLV-2 and HIV-1, *n* = 24	AIDS-defining pathologies were more frequently observed in the coinfected patients: 9/19(47%) vs. 30/128(23%) *p* = 0.02.	Similar Initial lymphocytes CD4 (cell/mL): 261 ± 232 vs. 202 ± 146, *p* = 0.4. Candidiasis more frequently in coinfected: 21/168 (12%) vs 10/24 (42 %), *p* = 0.0004
Collins (22)	Case control, Peru	Total *n* = 150 Male: 121 (87%) Age: m = 41 (± 11)	HTLV-1 and HIV-1, *n* = 50	Survival: 47 months (range = 17–77) vs. 85 months (range = 70–100) *p =* 0.06. Death rate: 7 (32%) vs. 7 (13%), [HR 1.6 (95% CI 1.0–2.8; *p* = 0.06)]. ART Use: 22/50 (44%) vs. 53/100 (53%), *p* = 0.5.	The variables associated to death were: Age > 40 years: HR Unadjusted = 1.8 (95% CI 1.1–3.0).-CD4+ <100 cell/μL, HR Unadjusted = 3.8 (95% CI 1.4–10.2).–AIDS, HR Adjusted = 13.5 (95% CI 1.4–132.3).-No HAART use, HR Adjusted = 96.5 (95% CI 17.0–546.3). AIDS clinical stage [HR: 13.5, 95% CI (1.4 −132.3)] and lack of antiretroviral therapy [HR: 96.5, 95% CI (17.0–546.3)], were associated to a higher risk of dying.
Pedroso (23)	Nested case control, Brazil	Total *n* = 74 Female: 39 (52.7%) Age: 2 to 16 years	HTLV-1/2 and HIV-1, *n* = 35	Mortality: 12/35 (34.3%) vs. 3/39 (7.7%), *p* = 0.01 Opportunistic: 88.6 vs. 44.7%, *p* = <0.001. –Shorter survival for coinfected patients, *p* = 0.003	The CD4+ cell count was higher in coinfected than in monoinfected patients: 1,429 ± 608 vs. 928 ± 768, *p* = 0.003. –Any clinical symptoms were described more frequently on coinfected (64.6%) than monoinfected patients (35.4%), OR: 9.6, 95% CI: (2.8–32.5).
Brites (24)	Retrospective, cross- sectional, Brazil	Total *n* = 123 Male: 97 (78.9%) Age: m = 33.4 (± 8.1)	HTLV-1/2 and HIV-1 *n* = 26	Strongyloidiasis: 4/26 (15.4%) vs. 2/97 (2.1%), [OR = 8.55; 95% CI: (1.21–73.62)], *p* = 0.02.	2 cases of encephalopathy were diagnosed in coinfected, vs. no case detected on monoinfected patients, *p* = 0.04. Coinfected patients were less frequently on ART use than monoinfected ones, (42.3 vs. 64.3%, *p* = 0.04).
Mendoza (25)	Cross- sectional, Spain	Total *n* = 369 Female: 227 (63.6%) Age: m = 50	HTLV-1 and HIV-1, *n* = 12	AIDS progression: 7/12(58%) vs. 10/357(2.8%). HAM/TSP: 2/12 (16%) vs. 46/357. (12.8%).	Frequency of clinical manifestations in coinfected patients was significantly higher than in monoinfected ones. The AIDS conditions reported on HTLV monoinfected patients were recurrent pneumonia, extrapulmonary tuberculosis, and esophageal candidiasis. Late diagnosis explains the high rate (9/12) of clinical manifestations in the HIV-HTLV co-infected population.
Brites (26)	Nested Retrospective Case-control, Brazil	Total *n* = 298 Female: 166 (59.1%) Age: m = 39.0 (±9.1)	HTLV-1 and HIV-1, *n* = 149	Survival: 16.7(±0.7) years vs. 18.1(±4) years, *p* = 0.001. Deaths: 53 (17.8%) vs. 23 (7.7%). Mortality rate 2.1 per 100 persons-year. - ART use: all participants (100%).	Shorter survival for coinfected patients with detectable pVL compared to those with undetectable viremia: ≥ 50 copies/mL: 8.4 ± 0.8 vs. 12.9 ± 1.4 years, *p* = 0.02 >1,000 copies/mL: 6.7 ± 0.9 vs. 11.0 ± 0.1 years, *p* = 0.04 Survival time did not differ for patients monoinfected (19.0 ± 0.4 years) vs. coinfected (20.2 ± 0.6 years) presenting with pVL <50 copies/mL (*P* = 0.5). Deaths were largely caused by AIDS-related conditions, and frequency of causes of death was similar across groups. Successful ART is able to normalize survival.

*HIV, Human Immunodeficiency Virus; HTLV, Human T-cell lymphotropic Virus; AIDS, Acquired Immunodeficiency Syndrome; WHO, World Health Organization; HAM/TSP, Human T-lymphotropic virus type-I-associated myelopathy / tropical spastic paraparesis; ART, Antiretroviral Therapy; PN, Peripheral Neuropathy; pVL, Plasma Viral Load; HR, Hazard Ratio*.

### Survival

Six articles describing survival were identified (8, 11, 15, 22, 23, 26). Four showed lower survival rate for HIV-1 and HTLV-1 or HTLV-1/2 coinfected subjects (8, 15, 23, 26), while the remaining studies found a neutral association between survival and HIV-1 and HTLV-2 coinfection status (11, 22).

### Progression to Death and Mortality

HIV-1 and HTLV-1 or HTLV 1/2 coinfected subjects were more likely to die than monoinfected ones, as shown in four studies (15, 19, 22, 26). However, Giacomo et al described that progression to death was slower in five HIV-1/HTLV-2 coinfected patients when compared with forty-four mono-infected ones (17). Death was largely caused by AIDS-related conditions and was associated with age > 40 years, CD4+ <100 cell/μL, and no ART use (22, 26). Brites found a higher mortality rate (17.8%) among HIV-1 and HTLV-1 coinfected adults on ART, while Pedroso described 34% of mortality in a pediatric HIV-1 and HTLV 1/ 2 coinfected population (23, 26). On the other hand, Hershow did not find any association between mortality and HIV-1 / HTLV-2 coinfection in 61 coinfected adults, and 23% of mono-infected vs. 20% of coinfected patients were on Zidovudine monotherapy, (*p* = 0.54) (18).

### AIDS or Opportunistic Diseases

HIV-1 and HTLV-1 coinfection was associated with AIDS progression in some studies and coinfection by these agents was also associated with a higher frequency of pneumonia, extrapulmonary tuberculosis, and esophageal candidiasis (7, 25). Coinfected patients also presented with a higher frequency of diseases like ichthyosis 8/36 (22%) vs. 12/148 (6.8%), *p* = 0.038 (16), crusted scabies 17 (18.6%) vs. 0 (0%), *p* = 0.01 (19), candidiasis 10 (42%) vs. 21 (12%), *p* = 0.0004 (21), and Strongyloidiasis 4/26 (15%) vs. 2/97 (2%), *p* = 0.02 (24) than monoinfected ones.

### Other Clinical Manifestations

Coinfected patients were more likely to present any clinical symptoms, 64.6 vs. 35.4%, OR: 9.6, 95% CI (2.8–32.5) (23, 25), central nervous system manifestations like encephalopathy 2 vs. 0, (*p* = 0.04) (24), peripheral neuropathy 20 (40%) vs. 8 (13.3%), *p* = 0.004 and HAM/TSP, 2/12 (16%) vs. 46/357 (12.8%) (20, 25) or more aggressive clinical manifestation like ichthyosis 8/36 (22.2%) vs. 12/148 (6.8%), *p* = 0.038, characterized by the scales larger, rhomboidal, extremely coarse, resembling those of lamellar ichthyosis, with also frequent palmoplantar keratoderma (16), and severe scabies, OR: 3.0; 95% CI (1.85–4.86) with crusted form being highly predictive of retroviral coinfection (19).

### HIV/AIDS Related Findings

CD4 cell count was consistently higher in coinfected patients as described also in Table 2 (7, 17, 23). In addition, one study reported that coinfected patients who died during the studies had higher CD4 T cells count than monoinfected ones 113 vs. 10 cells, (*p* = 0.023) (18). Survival was shorter for coinfected patients with detectable plasma viremia 6.7 ± 0.9 in comparison to those with undetectable viral load 11.0 ± 0.1 years (*p* = 0.04) (26). ART use was less frequent on coinfected patients when compared to monoinfected ones as follows: 42 vs. 64%, (*p* = 0.04) (24), 16/30 (53.3%) vs. 49/60 (81.6%) (20), 5 vs. 0% (*p* = 0.58) (7), 44 vs. 53% (*p* = 0.5) (22), but in two studies there was not statistical difference. In Zehender et al. study no patient with peripheral polyneuropathy was on ART at the moment of symptoms onset (20).

## Discussion

The aim of this systematical review was to describe the published information about clinical outcomes in HIV-1 and HTLV-1/HTLV-2 co-infected individuals from 1990 to October 2021. The available studies are controversial, methodologically heterogeneous and fail to identify the factors driving the outcomes observed in HIV-HTLV coinfected population. Although the present review lacks important information due to design of included studies, we were able to detect some relevant findings associated to coinfection.

HTLV-1 mono-infection can cause severe diseases in ~10% of infected individuals, including adult T-cell leukemia, infective dermatitis and HAM/TSP (27, 28). It is also associated with an increase in all-cause mortality, inflammatory and infectious conditions, and other cancers than ATL (27). On the other hand, HTLV-2 mono-infection is probably associated with HAM/TSP like disease, and some bacterial or parasitic infection (28). According to the findings described on this review similar outcomes are detected in HIV-1 / HTLV-1 coinfected patients, as well as in triple coinfection in HIV-1 and HTLV 1/2, resulting in a shorter survival/ higher mortality rates and faster AIDS progression (7, 8, 15, 19, 21, 23, 25, 26). Collins et al. showed similar results, but without reaching statistical significance (*p* = 0.06), despite describing that AIDS clinical stage [HR: 13.5, 95% CI (1.4–132.3)] and lack of antiretroviral therapy [HR: 96.5, 95% CI (17.0–546.3)], were associated to a higher risk of dying in HIV-1 and HTLV-1 coinfected ones (22). Although HIV-1 and HTLV-2 coinfection has been associated with longer survival, slower AIDS progression, and lower mortality rates (as described in Table 2), the reports describing a potential protective effect of HIV-1 and HTLV-2 coinfection against disease progression found a neutral association, and the available studies have clear limitations, due to the small number of participants, which makes difficult to reach reliable conclusions (11, 16, 22).

Higher rates of neurological manifestations were found in coinfected individuals, especially the myelopathy (HAM/TSP) related to HIV-1/HTLV-1 coinfection) and peripheral neuropathy (PN, in association with either HIV-1/HTLV-1 or HIV-1/HTLV-2 coinfection). HAM/TSP like manifestations was also described among HIV-1/HTLV-2 coinfection (29). Such findings are compatible with those reported by Zehender, who detected a higher frequency of peripheral neuropathy in HIV-1/HTLV-2 coinfection. Brites also reported cases of encephalopathy among HIV-1 and HTLV 1 co-infected patients, while Mendoza found a higher percentage of HAM/TSP in HIV-1/HTLV-1 co-infected patients (24, 25, 30). However, we found only a few clinical and neurological studies in coinfection, most of them describing small samples and having a short follow-up.

The rate of HIV disease progression may be affected by many factors, like the infecting viral strain, host susceptibility and immune function, as well as, to exogenous influences such as access to healthcare and presence of coinfections (31, 32). Our review shows that AIDS-defining conditions were more frequently seen on coinfected groups (16, 19, 21, 24, 25). Some published clinical studies are contradictory when describing the impact of HTLV-1 on AIDS evolution, but most of available evidence suggest that coinfection by HTLV-1 can modify the clinical course of HIV infection and that HIV also can promote a higher risk of HTLV-1 associated diseases (6, 9, 33, 34).

Several reports concluded that HIV/HTLV-1 co-infected patients show an increase in CD4+T cells count in comparison with HIV mono-infected ones, although there is no clear benefit in terms of immune response (7, 9, 17, 23, 24, 35). It is likely that a rise in dysfunctional CD4+T lymphocytes due to lymphoproliferative effect of HTLV- 1 would be responsible for such findings, leading to a false sense of immune competence in HIV co-infected individuals that could cause a delay in starting ART, when therapy initiation is guided by CD4 counts (25). On the other hand, in the coinfection by HTLV-2, the results consistently indicate no modification on the natural course of disease, and even a protective role to AIDS progression (9, 33). This effect may be the result of maintaining normal range levels of CD4 and CD8 counts, lowering HIV replication and immune activation (6). In addition, while HTLV-1 has tropism to CD4+ T cells, HTLV-2 is tropic to CD8+ T cells, which can explain part of the different outcomes observed in HTLV-1 and HTLV-2 infections. Furthermore, it is widely recognized that high viral load is strongly associated with faster progression to AIDS in HIV monoinfected subjects (36). The survival normalization observed in HIV/HTLV-1 coinfected subjects with suppressed plasma viremia, and the predominance of AIDS-related conditions as a cause of death in coinfected patients indicates that uncontrolled HIV infection is likely to be the main cause of worse prognosis observed in HIV-1 and HTLV-1 coinfected patients. The overestimation of immune status of coinfected patients due to the increase in CD4+ cells could lead, in the past, to a delay in starting ART. However, most of the current clinical guidelines for adults living with HIV AIDS recommend starting ART regardless of CD4 T-cell count (26). The reasons for the observed absence of significant impact of HTLV-2 coinfection on HIV infection are still poorly understood.

## Limitations

This review has some limitations: the findings described by the selected articles were observed in only 5 countries (USA, Italy, Brazil, Mexico and Perú), which limits the generalization to other populations in prevalent regions like, Australia and Japan. Most studies included middle-aged, and only one study was focused on a pediatric population limiting any conclusion related to this age group (23). Finally, we could not perform a direct comparison between studies due to their high methodological heterogeneity.

## Conclusions

HIV-1 and HTLV-1 coinfection and HIV-1 and HTLV-1/2 triple infection are related to lower survival rate, death, and faster progression to death, while coinfection by HIV-1/HTLV-2 seems have neutral associations with higher survival rate, slower AIDS progression, and lower mortality rate. AIDS defining conditions, opportunistic and neurological manifestations were more frequently described on HIV-HTLV-1 coinfected subjects, which indicates AIDS condition as the main cause of death for them. The implementation of test-and-treat strategy for patients living with HIV in areas endemic for HTLV infection is mandatory, to avoid the risks of delayed therapy and death for coinfected patients.

## Data Availability Statement

The raw data supporting the conclusions of this article will be made available by the authors, without undue reservation.

## Author Contributions

IM-C, CM, and CS: conception and design of the study, acquisition of data, analysis and interpretation of data, and drafting the article. CB: conception and design of the study, analysis and interpretation of data, drafting the article, and final approval of the version to be submitted. All authors agree to be accountable for the content of the work. All authors contributed to the article and approved the submitted version.

## Funding

IM-C has a CAPES (Coordenação de Aperfeiçoamento de Pessoal de Nível Superior) Ph.D. scholarship, finance Code 001. CM has a CNPq (Conselho Nacional de Desenvolvimento Ciente Tecnológico) Ph.D. scholarship, process number: 140187/2021-9. CB is a CNPq researcher, process number: 311095/2020-8. The funder had no role in study design, data collection, data analysis, data interpretation, and writing of the report.

## Conflict of Interest

The authors declare that the research was conducted in the absence of any commercial or financial relationships that could be construed as a potential conflict of interest.

## Publisher's Note

All claims expressed in this article are solely those of the authors and do not necessarily represent those of their affiliated organizations, or those of the publisher, the editors and the reviewers. Any product that may be evaluated in this article, or claim that may be made by its manufacturer, is not guaranteed or endorsed by the publisher.

## Nomenclature

HIV (Human Immunodeficiency Virus); HTLV (Human T-cell lymphotropic Virus); AIDS (Acquired Immunodeficiency Syndrome); WHO (World Health Organization); HAM/TSP (Human T-lymphotropic virus type-I-associated myelopathy/tropical spastic paraparesis); ART (Antiretroviral Therapy); PN (Peripheral Neuropathy); pVL (Plasma Viral Load); HR (Hazard Ratio).

## References

[1] Poiesz BJ Ruscetti FW Gazdar AF Bunn PA Minna JD Gallo RC. Detection and isolation of type C retrovirus particles from fresh and cultured lymphocytes of a patient with cutaneous T-cell lymphoma. Proc Nat Acad Sci. (1980) 77:7415–9. 10.1073/pnas.77.12.7415 6261256 PMC350514

[2] Osame M Usuku K Izumo S Ijichi N Amitani H Igata A . associated myelopathy, a new clinical entity. Lancet. (1986) 327:1031–2. 10.1016/S0140-6736(86)91298-5 2871307

[3] Lucas S Nelson AM. HIV and the spectrum of human disease. J Pathol. (2015) 235:229–41. 10.1002/path.4449 25251832

[4] Pilotti E Bianchi M V De Maria A Bozzano F Romanelli MG Bertazzoni U . HTLV-1/-2 and HIV-1 co-infections: retroviral interference on host immune status. Front Microbiol. (2013) 4:1–13. 10.3389/fmicb.2013.00372 24391628 PMC3870298

[5] Beilke MA. Retroviral coinfections: HIV and HTLV: Taking stock of more than a quarter century of research. AIDS Res Hum Retroviruses. (2012) 28:139–47. 10.1089/aid.2011.0342 22171689 PMC3275926

[6] Casoli C Pilotti E Bertazzoni U. Molecular and cellular interactions of HIV-1/HTLV coinfection and impact on AIDS progression. AIDS Rev. (2007) 9:140–9. 17982939

[7] Schechter M Harrison LH Halsey NA Trade G Santino M Moulton LH . Coinfection with human T-cell lymphotropic virus type I and HIV in Brazil. Impact on markers of HIV disease progression. JAMA. (1994) 271:353–7. 10.1001/jama.1994.03510290035033 7904317

[8] Brites C Alencar R Gusmão R Pedroso C Netto EM Pedral-Sampaio D . Co-infection with HTLV-1 is associated with a shorter survival time for HIV-1-infected patients in Bahia, Brazil. AIDS. (2001) 15:2053–5. 10.1097/00002030-200110190-00023 11600839

[9] Beilke MA Theall KP Megan O Clayton JL Benjamin SM Winsor EL . Clinical Outcomes and Disease Progression among Patients Coinfected with HIV and Human T Lymphotropic Virus Types 1 and 2. Clin Infect Dis. (2004) 39:256–63. 10.1086/422146 15307036

[10] Chavance M Neisson-Vernant C Quist D Monplaisir N Armengaud B Chout R. HIV/HTLV-I coinfection and clinical grade at diagnosis. J Acquir Immune Defic Syndr Hum Retrovirol. (1995) 8:91–5. 10.1097/00042560-199501000-00013 8548352

[11] Visconti A Visconti L Bellocco R Binkin N Colucci G Vernocchi L . HTLV-II/HIV-1 coinfection and risk for progression to AIDS among intravenous drug users. J Acquir Immune Defic Syndr. (1993) 6:1228–37. 7901382

[12] Page MJ McKenzie JE Bossuyt PM Boutron I Hoffmann TC Mulrow CD . The PRISMA 2020 statement: an updated guideline for reporting systematic reviews. BMJ. (2021) 372:n71. 10.1136/bmj.n71 33782057 PMC8005924

[13] Ouzzani M Hammady H Fedorowicz Z Elmagarmid A. Rayyan—a web and mobile app for systematic reviews. Syst Rev. (2016) 5:210. 10.1186/s13643-016-0384-4 27919275 PMC5139140

[14] Wells G Shea B Robertson J Peterson J Welch V Losos M . The Newcastle-Ottawa Scale (NOS) for Assessing the Quality of Nonrandomized Studies in Meta-Analysis. Available online at: http://www.ohri.ca/programs/clinical_epidemiology/oxford.asp (accessed November 15, 2021).

[15] Page JB Lai SH Chitwood DD Klimas NG Smith PC Fletcher MA. HTLV-I/II seropositivity and death from AIDS among HIV-1 seropositive intravenous drug users. Lancet. (1990) 335:1439–41. 10.1016/0140-6736(90)91456-K 1972217

[16] Kaplan MH Sadick NS McNutt NS Talmor M Coronesi M Hall WW. Acquired ichthyosis in concomitant HIV-1 and HTLV-II infection: a new association with intravenous drug abuse. J Am Acad Dermatol. (1993) 29:701–8. 10.1016/0190-9622(93)70234-K 8227542

[17] Giacomo M Franco EG Claudio C Carlo C Anna DA Anna D . Human T-cell leukemia virus type II infection among high risk groups and its influence on HIV-1 disease progression. Eur J Epidemiol. (1995) 11:527–33. 10.1007/BF01719304 8549726

[18] Hershow RC Galai N Fukuda K Graber J Vlahov D Rezza G . An international collaborative study of the effects of coinfection with human T-lymphotropic virus type II on human immunodeficiency virus type 1 disease progression in injection drug users. J Infect Dis. (1996) 174:309–17. 10.1093/infdis/174.2.309 8699060

[19] Brites C Weyll M Pedroso C Badaró R. Severe and Norwegian scabies are strongly associated with retroviral (HIV-1/HTLV-1) infection in Bahia, Brazil. AIDS. (2002) 16:1292–3. 10.1097/00002030-200206140-00015 12045498

[20] Zehender G Colasante C Santambrogio S De Maddalena C Massetto B Cavalli B . Increased Risk of Developing Peripheral Neuropathy in Patients Coinfected With HIV-1 and HTLV-2. J Acquir Immune Defic Syndr. (2002) 31:440–7. 10.1097/00126334-200212010-00011 12447016

[21] Castro-Sansores CJ Santos-Rivero A González-Martínez P Lara-Perera DM Alonso-Salomon G Gongora-Biachi RA. Co-Infection by the Human T-Cell Lymphotropic Virus Type II in Patients Infected by the Human Immunodeficiency Virus in Yucatan, Mexico. Arch Med Res. (2006) 37:365–9. 10.1016/j.arcmed.2005.06.013 16513486

[22] Collins JA Hernández A V Hidalgo JA Salazar R. HTLV- I infection is not associated with a higher risk of death in peruvian HIV-infected patients. Rev Inst Med Trop São Paulo. (2009) 51:197–201. 10.1590/S0036-46652009000400004 19738999

[23] Pedroso C Netto E Weyll N Brites C. Coinfection by HIV-1 and Human Lymphotropic Virus Type 1 in Brazilian Children Is Strongly Associated With a Shorter Survival Time. J Acquir Immune Defic Syndr. (2011) 58:e120. 10.1097/QAI.0b013e31821e9baf 21857320

[24] Brites C Goyanna F França L Pedroso C Netto E Adriano S . Coinfection by HTLV-I/II is associated with an increased risk of strongyloidiasis and delay in starting antiretroviral therapy for AIDS patients. Braz J Infect Dis. (2011) 15:6–11. 10.1016/S1413-8670(11)70132-5 21412582

[25] Mendoza C Caballero E Aguilera A Benito R Maciá D García-Costa J . co-infection in HTLV-1 carriers in Spain. Virus Res. (2019) 266:48–51. 10.1016/j.virusres.2019.04.004 30998953

[26] Brites C Miranda F Luz E Netto EM. Early and Successful Combination Antiretroviral Therapy Normalizes Survival Time in Patients Coinfected with Human Immunodeficiency Virus and Human T-cell Lymphotrophic Virus Type 1. Clin Infect Dis. (2020) 71:196–200. 10.1093/cid/ciz756 31406994

[27] Gonçalves DU Proietti FA Ribas JGR Araújo MG Pinheiro SR Guedes AC . Epidemiology, Treatment, and Prevention of Human T-Cell Leukemia Virus Type 1-Associated Diseases. Clin Microbiol Rev. (2010) 23:577–89. 10.1128/CMR.00063-09 20610824 PMC2901658

[28] Nicolás D Ambrosioni J Paredes R Marcos MÁ Manzardo C Moreno A . Infection with human retroviruses other than HIV-1: HIV-2, HTLV-1, HTLV-2, HTLV-3 and HTLV-4. Expert Rev Anti Infect Ther. (2015) 13:947–63. 10.1586/14787210.2015.1056157 26112187

[29] Araujo AQ-C. Neurological Aspects of HIV-1/HTLV-1 and HIV-1/HTLV-2 Coinfection. Pathogens. (2020) 9:250. 10.3390/pathogens9040250 32231144 PMC7238008

[30] Zehender G Meroni L Varchetta S De Maddalena C Cavalli B Gianotto M . Human T-lymphotropic virus type 2 (HTLV-2) provirus in circulating cells of the monocyte/macrophage lineage in patients dually infected with human immunodeficiency virus type 1 and HTLV-2 and having predominantly sensory polyneuropathy. J Virol. (1998) 72:7664–8. 10.1128/JVI.72.9.7664-7668.1998 9696872 PMC110036

[31] Lawn SD Butera ST Folks TM. Contribution of Immune Activation to the Pathogenesis and Transmission of Human Immunodeficiency Virus Type 1 Infection. Clin Microbiol Rev. (2001) 14:753–77. 10.1128/CMR.14.4.753-777.2001 11585784 PMC89002

[32] Harrer T Harrer E Kalams SA Elbeik T Staprans SI Feinberg MB . Strong cytotoxic T cell and weak neutralizing antibody responses in a subset of persons with stable nonprogressing HIV type 1 infection. AIDS Res Hum Retroviruses. (1996) 12:585–92. 10.1089/aid.1996.12.585 8743084

[33] Brites C Sampalo J Oliveira A. HIV/human T-cell lymphotropic virus coinfection revisited: impact on AIDS progression. AIDS Rev. (2009) 11:8–16. 19290030

[34] Isache C Sands M Guzman N Figueroa D. HTLV-1 and HIV-1 co-infection: a case report and review of the literature. IDCases. (2016) 4:53–5. 10.1016/j.idcr.2016.03.002 27144124 PMC4840448

[35] Ticona E Huaman MA Yanque O Zunt JR. HIV and HTLV-1 Coinfection: the need to initiate antiretroviral therapy. J Int Assoc Provid AIDS Care. (2013) 12:373–4. 10.1177/2325957413500988 24222069 PMC4170712

[36] Mellors JW Rinaldo CR Gupta P White RM Todd JA Kingsley LA. Prognosis in HIV-1 Infection Predicted by the Quantity of Virus in Plasma. Science. (1996) 272:1167–70. 10.1126/science.272.5265.1167 8638160

